# Income-related health inequality among Chinese adults during the COVID-19 pandemic: evidence based on an online survey

**DOI:** 10.1186/s12939-021-01448-9

**Published:** 2021-04-26

**Authors:** Peng Nie, Lanlin Ding, Zhuo Chen, Shiyong Liu, Qi Zhang, Zumin Shi, Lu Wang, Hong Xue, Gordon G. Liu, Youfa Wang

**Affiliations:** 1 School of Economics and Finance, Xi’an Jiaotong University, Xi’an, 710061 Shaanxi China; 2 Global Health Institute, School of Public Health, Xi’an Jiaotong University Health Science Center, Xi’an, 710061 Shaanxi China; 3 Department of Health Policy and Management, College of Public Health, University of Georgia, Athens, GA USA; 4 School of Economics, Faculty of Humanities and Social Sciences, University of Nottingham Ningbo China, Ningbo, China; 5 Center for Governance Studies, Beijing Normal University at Zhuhai, Zhuhai, 519087 China; 6 School of Community and Environmental Health, Old Dominion University, Norfolk, VA USA; 7 Human Nutrition Department, College of Health Sciences, QU Health, Qatar University, Doha, Qatar; 8 Department of Health Administration and Policy, College of Health and Human Services, George Mason University, Fairfax, VA 22030 USA; 9 Peking University National School of Development, Beijing, 100871 China

**Keywords:** COVID-19, Health inequality, Mental health, Socioeconomic status, China

## Abstract

**Background:**

Partial- or full-lockdowns, among other interventions during the COVID-19 pandemic, may disproportionally affect people (their behaviors and health outcomes) with lower socioeconomic status (SES). This study examines income-related health inequalities and their main contributors in China during the pandemic.

**Methods:**

The 2020 China COVID-19 Survey is an anonymous 74-item survey administered via social media in China. A national sample of 10,545 adults in all 31 provinces, municipalities, and autonomous regions in mainland China provided comprehensive data on sociodemographic characteristics, awareness and attitudes towards COVID-19, lifestyle factors, and health outcomes during the lockdown. Of them, 8448 subjects provided data for this analysis. Concentration Index (CI) and Corrected CI (CCI) were used to measure income-related inequalities in mental health and self-reported health (SRH), respectively. Wagstaff-type decomposition analysis was used to identify contributors to health inequalities.

**Results:**

Most participants reported their health status as “very good” (39.0%) or “excellent” (42.3%). CCI of SRH and mental health were − 0.09 (*p < 0.01*) and 0.04 (*p < 0.01*), respectively, indicating pro-poor inequality in ill SRH and pro-rich inequality in ill mental health. Income was the leading contributor to inequalities in SRH and mental health, accounting for 62.7% (*p < 0.01*) and 39.0% (*p < 0.05*) of income-related inequalities, respectively. The COVID-19 related variables, including self-reported family-member COVID-19 infection, job loss, experiences of food and medication shortage, engagement in physical activity, and five different-level pandemic regions of residence, explained substantial inequalities in ill SRH and ill mental health, accounting for 29.7% (*p < 0.01*) and 20.6% (*p < 0.01*), respectively. Self-reported family member COVID-19 infection, experiencing food and medication shortage, and engagement in physical activity explain 9.4% (*p < 0.01*), 2.6% (the summed contributions of experiencing food shortage (0.9%) and medication shortage (1.7%), *p < 0.01*), and 17.6% (*p < 0.01*) inequality in SRH, respectively (8.9% (*p < 0.01*), 24.1% (*p < 0.01*), and 15.1% (*p < 0.01*) for mental health).

**Conclusions:**

Per capita household income last year, experiences of food and medication shortage, self-reported family member COVID-19 infection, and physical activity are important contributors to health inequalities, especially mental health in China during the COVID-19 pandemic. Intervention programs should be implemented to support vulnerable groups.

**Supplementary Information:**

The online version contains supplementary material available at 10.1186/s12939-021-01448-9.

## Introduction

Disease pandemic is one of the leading health threats worldwide [1]. As of March 8, 2021, confirmed cases of the novel coronavirus disease (COVID-19) exceeded 116 million, with approximately 2.6 million deaths across 216 countries and regions [2]. On the same day, China reported 102,101 accumulated cases and 4848 deaths [2]. Due to the COVID-19’s contagiousness and relatively high case mortality rate, the pandemic has a profound impact on all aspects of the society [3–5]. In addition to the direct impact on population health, the negative economic fallout of the pandemic had emerged [4].

The nature of the economic and policy responses to the COVID-19 pandemic has created gradients in exposure not only to the disease itself but also to the economic consequences of the lockdown [6]. As such, the COVID-19 pandemic has intensified existing SES-related inequalities in health and may exacerbate such inequalities by impacting vulnerable populations far more than their better-off counterparts [6, 7]. Since January 2020, China has implemented various containment measures, including community quarantine, self-isolation, and social distancing; while differences exist in the practices across regions in China due to their specific situation related to the number of cases reported, social and economic development levels, etc. These measures, together with the economic impacts of the partial shutdown of the economy, have accentuated the mental health problems of the affected population [8]. Considering the characteristics of the policy and institutional responses to COVID-19, the burden of this pandemic may have been unequally distributed across the population.

A growing body of literature has explored the extent of inequalities and the relationship between SES and health inequality. Using Concentration Index (CI) method to measure income-related health inequalities in mental health in the UK, a study reported substantial inequality unfavorable to low-income groups [9]. Another study confirmed the existence of income-related health inequalities across Europe and showed that income inequality was the dominant contributor to health inequalities [10]. Meanwhile, studies in China had confirmed the existence of pro-rich inequalities in various health outcomes such as self-reported health (SRH) [11–15], health-related quality of life [16], physical-activity (PA) limitations [12], maternal mortality [17], and high blood pressure [18]. Variables such as age, gender, education, and health-related behaviors have been identified as important sources of health inequalities [12, 16, 19]. Determinants such as age and gender are not amenable to changes, whereas others such as education and health-related behaviors are modifiable; thus the related inequalities avoidable [19]. Health inequalities associated with modifiable factors are a form of health inequity, and it is necessary to eliminate or attenuate the inequalities via targeted policy interventions. Although the COVID-19 pandemic may have exacerbated pre-existing SES-related health inequalities while generating new forms of disparities [4, 6], few studies have quantified income-related health inequalities and explored their sources in the context of the COVID-19 pandemic, with a focus on COVID-19 exposure or experiences.

Using data from a nationwide survey in China, this study estimates income-related health inequalities and explores the contributions to the inequalities of sociodemographic and COVID-19 related characteristics, including COVID-19 infection, job loss, experiences of food or medication shortage, engagement in PA, and five-level pandemic severity in the province of residence. Our study contributes to the understanding of income-related health inequalities during the COVID-19 pandemic and facilitates policy and program development to support vulnerable populations during the pandemic.

## Methods and materials

### Study design and participants

The data were drawn from the “2020 China COVID-19 Survey”, an anonymous cross-sectional survey administered via WeChat (China’s leading messaging and social networking mobile APP with the monthly active user exceeding one billion since 2018) [20–22]. Responses were collected between late April and early May 2020. We used both snowball and convenience sampling to recruit a diverse national sample across China.

The 2020 China COVID-19 Survey questionnaire has 74 items and contains 150 study variables. It encompasses eight topics: (1) awareness, attitude, knowledge, and practices toward COVID-19, (2) COVID-19 experiences and impacts, (3) attitude towards government responses to COVID-19, (4) healthcare-seeking behaviors, (5) demographic characteristics, (6) lifestyle behaviors, (7) psychological well-being, and (8) health outcomes including obesity and other chronic diseases during the COVID-19 pandemic. Data include a national sample of 10,545 adults aged 18 years and over in all 31 province-level administrative units in China. The Institutional Review Board at Xi’an Jiaotong University approved the study procedures. Participants provided informed consent online. The subjects of this analysis are limited to 8448 adults aged 18 and older with complete data.

### Measurements

#### Health variables

In this study, we included SRH and mental health as the main health outcomes. SRH is an ordinal variable measured on 5-point scale, with 1 = excellent, 2 = very good, 3 = good, 4 = fair, and 5 = poor. Because few respondents reported “fair” and “poor” categories (0.24% reported “poor” and 2.69% reported “fair”), we combined those two groups into one as “poor/fair.” We measured mental health based on responses to screening items of the widely used and validated civilian version of the posttraumatic stress disorder checklist [23], asking respondents whether, during the past month, they had felt 1) Anhedonia: loss of interest in activities you liked in the past, 2) Sleep problems: difficulty falling asleep, or staying asleep, or waking up frequently or early, 3) Anger: being easily irritable or angry, 4) Difficulty in concentrating, or 5) Repeated disturbing dreams related to COVID-19. Respondents indicate the frequency of each feeling on a 5-point scale of 1 = not at all, 2 = a little, 3 = some, 4 = a lot, and 5 = extremely. We generated a composite score of mental health by summing the values for all five responses, which yielded a total score between 5 and 25 with higher values indicating more mental health problems.

#### Independent variables

Independent variables are categorized into four groups:

Sociodemographic characteristics: we included age, gender, education (low = “elementary school or below”, medium = “junior high/high school diploma/some college/associated degree,” and high = “bachelor’s degree/master’s degree or above”), marital status (unmarried, married/cohabiting, or divorced/separated/widowed), employment status (unemployed, employed, student, or retired), per capita annual household income in 2019, and residence (rural, town, or city). Towns in China typically include an urban central business district and a surrounding rural area with scattered villages. Compared with cities, towns are relatively small in size and population.

Noncommunicable chronic diseases (NCDs) of respondents: NCDs included high blood pressure, diabetes, heart disease, stroke, tumor/cancer, asthma, chronic lung disease, chronic kidney disease, liver disease, and compromised immune system. We added the number of NCDs that the respondent suffered from (measured on a 4-point scale, with 0, 1, 2, and ≥ 3).

Health-related lifestyles and medical insurance: we included alcohol drinking (none, ex-drinker, or current drinker), and smoking (none, ex-smoker, or current smoker). We also added knowledge of the Chinese Dietary Pagoda, indicating whether the respondent has heard of the “Chinese Dietary Guideline” (1 = yes and 0 = no). Insurance status indicates whether the respondent has medical insurance (1 = yes and 0 = no).

COVID-19 related variables: we added six variables related to COVID-19, including whether the respondent or his/her family lost job(s) due to COVID-19, had COVID-19 infection in the family, experienced food shortage, or medication shortage, engaged in PA during COVID-19, and a five-level category indicating the pandemic severity in the province of residence. The category ranges from Level 1 to 5, with a higher level indicating a less severity of COVID-19 pandemic. Specifically, Level 1 (the cumulative number of confirmed cases (*N*) by February 20, 2020 ≥ 10,000) included Hubei province. Level 2 (1000 ≤ *N* < 10,000) included Guangdong and Zhejiang provinces. Level 3 (500 ≤ *N* < 1000) encompassed Henan, Hunan, Anhui and Jiangxi provinces. Level 4 (100 ≤ *N* < 500) included Jiangsu, Chongqing, Shandong, Sichuan, Beijing, Heilongjiang, Shanghai, Fujian, Shaanxi, Hebei, Guangxi, Yunnan, and Hainan provinces. Level 5 (*N* < 100) included Liaoning, Shanxi, Tianjin, Jilin, Inner Mongolia, Guizhou, Tibet, Gansu, Ningxia, Xinjiang, and Qinghai provinces. A detailed definition is available in Additional file 1.

### Statistical analysis

First, we assumed a multiple linear and additive regression model to explore the determinants of health status *h*_*i*_:

1 $$ {h}_i=\alpha +{\sum}_{k=1}^K{\beta}_k{x}_{ik}+{\varepsilon}_i $$

where *x*_*ik*_ (*k* = 1, …, *K*) are the independent variables for individual *i*, *β*_*k*_ denotes the coefficient of *x*_*ik*_, and *ε*_*i*_ the error term. Because mental health scores are continuous, we used OLS regression. As SRH is ordinal, we used the ordered logit model.

Second, we used the CI to measure income-related health inequalities [24]. The CI is defined as twice the area between the concentration curve and the line of equality, ranging from − 1 to 1 (Additional file 7) [25]. Health endowments are equally distributed when the CI equals 0. If the health outcome is a “good,” a positive value of CI represents the existence of pro-rich inequality, meaning health endowments are concentrated among the rich. However, a negative value of CI denotes pro-poor inequality, i.e., health endowments are concentrated among the poor. The CI is expressed as:

2 $$ CI=\frac{2}{\mu}\mathit{\operatorname{cov}}\left({h}_i,{r}_i\right) $$

where *h*_*i*_ is the health status, *μ* is its mean, *r*_*i*_ is the fractional rank of individual *i* in the household income distribution.

Given that SRH is ordinal, we assumed individual health status is a latent cardinal variable. Therefore, we used the re-scaled predicted linear index of an ordered logit model (${h}_i^{\ast }$) as individual health [15, 26]. To calculate CI, the prediction from the ordered logit model can be re-scaled to the [0, 1] interval:

3 $$ {h}_i^{\ast }=\left({h}_i^1-{h}^{min}\right)/\left({h}^{max}-{h}^{min}\right) $$

where ${h}_i^1$ is the predicted linear index, and *h*^*min*^ and *h*^*max*^ are minimum and maximum of ${h}_i^1$, respectively [15, 27]. The use of continuous re-scaled latent SRH and mental health score can mitigate the index’s sensitivity to the numerical scale for categorical variables [28].

CI requires that health variables be measured on the same scale as income, namely, a ratio-scale without an upper bound [26, 29]. However, health variables are likely to be bounded and either ordinal or cardinal. Since both mental health and re-scaled predicted linear index for SRH are bounded, the common CI will estimate the inequality improperly. Thus, we use the corrected CI (CCI, defined as 4*μ*/(*b* − *a*) ∗ *CI*) [26]. Since higher values denote worse health status for both continuous mental health score and re-scaled latent SRH, we calculate the ill-health CI/CCI, i.e., income-related inequalities in ill health.

Linearity is a useful property for CI decomposition [27]. The continuous mental health score and re-scaled latent SRH are desirable for decomposition. Using Wagstaff decomposition, we decomposed CI of *h*_*i*_ as [24]:

4 $$ CI={\sum}_{k=1}^K\left({\beta}_k{\overline{x}}_k/\mu \right){CI}_k+\delta $$

where ${\overline{x}}_k$ is the mean of the independent variable *x*_*k*_, *CI*_*k*_ is the CI of *x*_*k*_, *δ* is the unexplained residual component. Thus, the CI equals a weighted sum of the CI of all independent variables *x*_*k*_, where the weight for *x*_*k*_ is the elasticity of *h*_*i*_ to *x*_*k*_ ($\eta ={\beta}_k{\overline{x}}_k/\mu$), and the unexplained residual part.

Based on the CI decomposition procedure, we partitioned CCI as follows:

5 $$ CCI=4{\sum}_{j=1}^k\left(\frac{\beta_k{\overline{x}}_k}{b-a}\right){CI}_k+\zeta $$

where *a* and *b* are the lower bound and upper bound for health outcomes, respectively. *ζ* is the unexplained residual component. Since the re-scaled latent SRH variable is a linear combination of regressors, there are no residuals included in ${h}_i^1$. Thus, in the decomposition analysis, we reported the contributions of each factor in the explained part of regressions (*conindex*).

All analyses were performed using STATA 16.1 (Stata Corporation, College Station, TX, USA).

## Results

### Study population characteristics

Our study sample covered 31 provinces/autonomous regions/municipalities in China. Table 1 shows that 39.0 and 42.3% of the respondents reported “very good” and “excellent” health, respectively. The mean mental health score was 10.6. The average age was 32.0 years old. The majority of respondents had medium- and high-level education (98.1%). On disease and health-related behaviors, 21.2% had one or more NCDs, 23.1% were current alcohol users, and 16.3% were current smokers. For COVID-19 related variables, 35.0% of respondents and their families lost jobs due to COVID-19, 28.3, and 31.1% experienced food and medication shortage, respectively, during the pandemic.

**Table 1:** Health and demographics among Chinese adults aged 18 years and older: The 2020 China COVID-19 Survey (*n* = 8448)

Variables	Total (*n* = 8448)	Female (*n* = 4747)	Male (*n* = 3701)	*p* value
	Mean (SD) /*N* (%)	Mean (SD) /*N* (%)	Mean (SD) /*N* (%)	
***Self-reported health (SRH)***				
Excellent	3577 (42.34%)	1818 (38.30%)	1759 (47.53%)	< 0.001
Very good	3296 (39.02%)	1982 (41.75%)	1314 (35.50%)	< 0.001
Good	1341 (15.87%)	798 (16.81%)	543 (14.67%)	0.008
Poor/fair	234 (2.77%)	149 (3.14%)	85 (2.30%)	0.019
Re-scaled latent SRH (${h}_i^{\ast }$)^a^	0.48 (0.13)	0.51 (0.13)	0.44 (0.13)	< 0.001
***Mental health***^*b*^	10.62 (4.97)	10.27 (4.79)	11.06 (5.17)	< 0.001
***Sociodemographic characteristics***				
Age (in years)	32.04 (9.97)	32.79 (9.92)	31.08 (9.96)	< 0.001
Education				
Low	163 (1.93%)	77 (1.62%)	86 (2.32%)	0.020
Middle	3479 (41.18%)	2035 (42.87%)	1444 (39.02%)	< 0.001
High	4806 (56.89%)	2635 (55.51%)	2171 (58.66%)	0.004
Employment status				
Unemployed	936 (11.08%)	654 (13.78%)	282 (7.62%)	< 0.001
Employed	5777 (68.38%)	3160 (66.57%)	2617 (70.71%)	< 0.001
Student	1464 (17.33%)	731 (15.40%)	733 (19.81%)	< 0.001
Retired	271 (3.21%)	202 (4.26%)	69 (1.86%)	< 0.001
Marital status				
Unmarried	2508 (29.69%)	1225 (25.81%)	1283 (34.67%)	< 0.001
Married/cohabiting	5774 (68.35%)	3414 (71.92%)	2360 (63.77%)	< 0.001
Divorced/separated/widowed	166 (1.96%)	108 (2.28%)	58 (1.57%)	0.020
Residence				
Rural	1282 (15.18%)	773 (16.28%)	509 (13.75%)	0.001
Town	2074 (24.55%)	1293 (27.24%)	781 (21.10%)	< 0.001
City	5092 (60.27%)	2681 (56.48%)	2411 (65.14%)	< 0.001
Per capita household income last year (categorical)				
Low (1st tertile)	3592 (42.52%)	2063 (43.46%)	1529 (41.31%)	0.048
Middle (2nd tertile)	2152 (25.47%)	1142 (24.06%)	1010 (27.29%)	< 0.001
High (3rd tertile)	2704 (32.01%)	1542 (32.48%)	1162 (31.40%)	0.288
***Chronic diseases (numbers)***				
Numbers of suffering from chronic diseases				
0	6657 (78.80%)	3880 (81.74%)	2777 (75.03%)	< 0.001
1	846 (10.01%)	455 (9.59%)	391 (10.56%)	0.137
2	505 (5.98%)	233 (4.91%)	272 (7.35%)	< 0.001
≥ 3	440 (5.21%)	179 (3.77%)	261 (7.05%)	< 0.001
***Lifestyles and medical insurance***				
Alcohol drinking				
None	5710 (67.59%)	3934 (82.87%)	1776 (47.99%)	< 0.001
Ex-drinker	785 (9.29%)	247 (5.20%)	538 (14.54%)	< 0.001
Current drinker	1953 (23.12%)	566 (11.92%)	1387 (37.48%)	< 0.001
Smoking				
None	6445 (76.29%)	4348 (91.59%)	2097 (56.66%)	< 0.001
Ex-smoker	623 (7.37%)	160 (3.37%)	463 (12.51%)	< 0.001
Current smoker	1380 (16.34%)	239 (5.03%)	1141 (30.83%)	< 0.001
Knowledge of Chinese Dietary Pagoda	6145 (72.74%)	3431 (72.28%)	2714 (73.33%)	0.280
Having medical insurance	7388 (87.45%)	4088 (86.12%)	3300 (89.17%)	< 0.001
***COVID-19 related variables***				
Losing job due to COVID-19	2958 (35.01%)	1461 (30.78%)	1497 (40.45%)	< 0.001
Self-reported family member COVID-19 infection	786 (9.30%)	302 (6.36%)	484 (13.08%)	< 0.001
Experiencing food shortage during COVID-19 lockdown	2389 (28.28%)	1106 (23.30%)	1283 (34.67%)	< 0.001
Experiencing medication shortage during COVID-19 lockdown	2629 (31.12%)	1241 (26.14%)	1388 (37.50%)	< 0.001
Engaging in any physical activity/exercise during COVID-19 lockdown	5283 (62.54%)	2787 (58.71%)	2496 (67.44%)	< 0.001
Pandemic severity in the province of residence^c^				
Level 1 pandemic severity	218 (2.58%)	104 (2.19%)	114 (3.08%)	0.011
Level 2 pandemic severity	684 (8.10%)	319 (6.72%)	365 (9.86%)	< 0.001
Level 3 pandemic severity	681 (8.06%)	362 (7.63%)	319 (8.62%)	0.096
Level 4 pandemic severity	4354 (51.54%)	2489 (52.43%)	1865 (50.39%)	0.063
Level 5 pandemic severity	2511 (29.72%)	1473 (31.03%)	1038 (28.05%)	0.003

The differences tests between female and male are based *t-test*, and *p-values* are reported

^a^ ${h}_i^{\ast }$ is the re-scaled predicted linear index of an ordered logit model, ranging from 0 to 1, with a higher value indicating worse SRH

^b^ In the survey, we collected the information on 1) Anhedonia: loss of interest in activities you liked in the past, 2) Sleep problems: difficulty falling asleep, or staying asleep, or waking up frequently or early, 3) Anger: got easily irritable or angry, 4) Difficulty concentrating, or 5) Repeated disturbing dreams related to COVID-19. Respondents indicate the frequency of each feeling on a 5-point scale of 1 = not at all, 2 = a little, 3 = some, 4 = a lot, and 5 = extremely. We generate a composite score of mental health by summing the values for all five responses, which yielded a total score between 5 and 25 with higher values indicating higher levels of mental health problems

^c^ The definition of 5-level pandemic severity in the province of residence is detailed in the Additional file 1

### Health status by income groups and income-related health inequality indexes

Table 2 presents the health status in different income groups (defined by income tertiles) and health inequality indexes of ill SRH and mental health. The average re-scaled ill SRH score for the low-income group was 0.52, whereas it was 0.49 and 0.43 for middle- and high-income groups. Similarly, the mental health scores for the low, middle, and high-income groups are 10.23, 10.38, and 11.32, respectively. High-income groups had better SRH but poorer mental health than middle- and low-income groups.

**Table 2:** Differences in health status of different income groups and inequality indexes of self-reported health and mental health among Chinese adults: The 2020 China COVID-19 Survey (*n* = 8448)

	Re-scaled latent SRH (${h}_i^{\ast }$)			Mental health		
	Mean ± SD^a^	CI	CCI	Mean ± SD^a^	CI	CCI
All		−0.0477^***^	−0.0920^***^		0.0204^***^	0.0433^***^
Income (low)	0.52 ± 0.13			10.23 ± 4.65		
Income (middle)	0.49 ± 0.13			10.38 ± 4.80		
Income (high)	0.43 ± 0.13			11.32 ± 5.42		
Female		−0.0524^***^	− 0.1014^***^		0.0154^***^	0.0316^***^
Income (low)	0.52 ± 0.13			9.94 ± 4.42		
Income (middle)	0.49 ± 0.14			10.17 ± 4.72		
Income (high)	0.43 ± 0.13			10.80 ± 5.24		
Male		−0.0472^***^	−0.0899^***^		0.0270^***^	0.0596^***^
Income (low)	0.51 ± 0.12			10.62 ± 4.91		
Income (middle)	0.49 ± 0.12			10.62 ± 4.89		
Income (high)	0.42 ± 0.13			12.01 ± 5.58		

Income is defined as income tertiles, with low (1st tertile), middle (2nd tertile), and high (3rd tertile)

*SRH* self-reported health, *CI* Concentration Index, *CCI* corrected CI

^***^ *p* < 0.01

^a^ Since we use the continuous re-scaled latent SRH and mental health score, we directly report their mean values

### Income-related health inequality

CCI of ill SRH and mental health were − 0.09 and 0.04, respectively, while some gender-differences existed. The CCI values of ill SRH and mental health were − 0.10 and 0.03 in females vs. − 0.09 and 0.06 in males, respectively. Results of CI and CCI calculations were consistent, indicating pro-rich inequality in SRH and pro-poor inequality in mental health.

### Decomposition of health inequality

Table 3 illustrates the decomposition of income-related health inequalities in SRH and mental health, respectively. According to Eq. (5), each column shows the regression coefficients, the CI for each variable, and the contribution of each factor to the explained inequalities. The income accounted for 62.7% of the contribution to inequalities in ill SRH (Table 3). The second-largest contribution (29.7%) to the income-related inequalities in SRH was from COVID-19 related variables, including job loss due to COVID-19, being affected by COVID-19, food and medication shortage, engagement in any PA, and pandemic severity in the province of residence. Engaging in PA and getting affected by COVID-19 were two major contributors, contributing 17.6 and 9.4% of the inequalities, respectively.

**Table 3:** Contribution of factors to income-related inequalities in ill SRH and mental health among Chinese adults: The 2020 China COVID-19 Survey (*N* = 8448)

	Ill SRH			Mental health		
	Coef.	CI_k_	Contribution^a^	Coef.	CI_k_	Contribution^a^
***Demographics***						
Gender	−0.0504^***^	0.0004	0.03%	−0.0697	0.0004	−0.01%
Age (in years)	0.0043^***^	0.0068	−4.10%	−0.0619^***^	0.0068	−9.18%
S***ocioeconomic status (SES)***						
Education						
Middle	0.0822^**^	0.0349	−5.14%	0.1601	0.0349	1.58%
High	0.1298^***^	−0.0265	8.50%	−0.0813	−0.0265	0.84%
Employment status						
Employed	−0.0478^***^	0.0389	5.53%	−0.1448	0.0389	−2.64%
Student	−0.0158	−0.1899	−2.26%	−0.5919^**^	−0.1899	13.32%
Retired	0.0020	0.0562	−0.02%	−0.5575^*^	0.0562	−0.69%
Marital status						
Married/cohabiting	−0.0450^***^	0.0537	7.19%	−0.3267^**^	0.0537	−8.21%
Divorced/separated/widowed	0.0402	0.0632	−0.22%	− 0.5271	0.0632	−0.45%
Residence						
Town	0.0137	−0.0403	0.59%	0.3205^*^	−0.0403	−2.17%
City	−0.0015	0.0363	0.14%	0.3087^**^	0.0363	4.62%
Per capita household income last year (continuous)	−0.0004^***^	0.7434	62.71%	0.0015^**^	0.7434	38.96%
***Chronic diseases (numbers)***						
1	0.1153^***^	−0.0106	0.53%	1.4365^***^	−0.0106	−1.04%
2	0.0844^***^	0.1582	−3.47%	2.4734^***^	0.1582	16.00%
≥ 3	0.0443^**^	0.2001	−2.01%	2.8981^***^	0.2001	20.66%
***Lifestyles***						
Alcohol drinking						
Ex-drinker	0.0076	0.0031	−0.01%	0.3418^*^	0.0031	0.07%
Currently drinker	0.0156	0.0392	−0.61%	0.2241	0.0392	1.39%
Smoking						
Ex-smoker	0.0039	0.0457	−0.06%	0.2191	0.0457	0.50%
Currently smoker	−0.0320^**^	0.1041	2.37%	0.5669^***^	0.1041	6.59%
Knowledge of dietary pagoda	−0.0359^***^	0.0077	0.88%	−0.5136^***^	0.0077	−1.97%
Medical insurance	−0.0192	−0.0034	−0.25%	−0.5918^***^	−0.0034	1.19%
***COVID-19 related variables***						
Losing job due to COVID-19	−0.0037	0.0268	0.15%	0.9685^***^	0.0268	6.22%
Self-reported family member COVID-19 infection	−0.1343^***^	0.1727	9.38%	0.8085^***^	0.1727	8.88%
Experiencing food shortage during COVID-19 lockdown	0.0121	0.0608	−0.90%	1.5199^***^	0.0608	17.89%
Experiencing medication shortage during COVID-19 lockdown	0.0283^***^	0.0448	−1.72%	0.6561^***^	0.0448	6.26%
Engaging in any physical activity/exercise during COVID-19 lockdown	−0.1540^***^	0.0420	17.57%	−0.8424^***^	0.0420	−15.12%
Pandemic severity in the province of residence						
Level 2 pandemic severity	−0.0529^**^	−0.0181	−0.34%	−0.4240	−0.0181	0.42%
Level 3 pandemic severity	−0.0431	−0.1050	−1.59%	−0.6717^**^	−0.1050	3.89%
Level 4 pandemic severity	−0.0846^***^	−0.0059	−1.11%	−0.8495^***^	−0.0059	1.76%
Level 5 pandemic severity	−0.1369^***^	0.0464	8.21%	−1.0136^***^	0.0464	−9.56%
Contribution of Covid-19 related variables			29.66%			20.64%
Total			100%			100%

*CI*_*k*_ Concentration Index of factor k

^*^ *p* < 0.1, ^**^ *p* < 0.05, ^***^ *p* < 0.01

^a^ Contribution (%) is defined as the contribution of each factor to the total explained part

Likewise, income remained the leading contributor to inequality in mental health, explaining 39.0% (Table 3). Being the second-largest contributor, NCDs accounted for 35.6% of the mental health inequality. The contribution of COVID-19 related variables to inequality in mental health was 20.6%. In particular, two major contributors were experiencing food shortages and engaging in PA during the pandemic. The experience of food shortage worsened inequality in mental health but engagement in PA mitigated inequality in mental health.

**Fig. 1:**
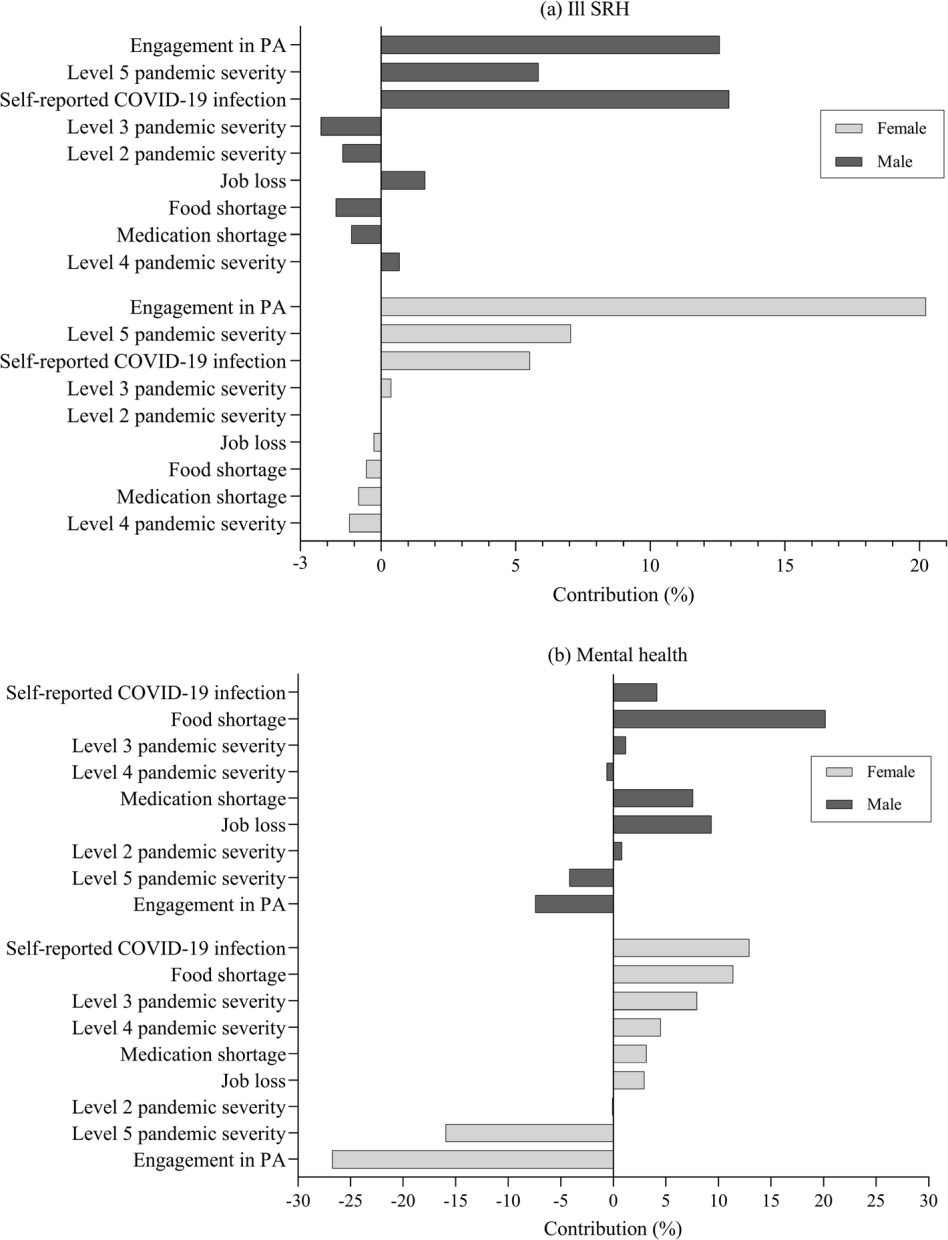
Contributions of COVID-19 related variables to inequality in SRH and mental health among Chinese adults

Additional files 2 and 3 present the decomposition of inequalities in SRH and mental health by gender. Figure 1 illustrates the contributions of COVID-19 related variables to inequalities in two health outcomes by gender. The summed contributions of COVID-19 factors to inequalities in ill SRH among females and males were 30.4 and 27.2%, respectively. In particular, PA during the COVID-19 pandemic was the dominant contributor, accounting for 20.2% among females and 12.6% among males (Fig. 1). In contrast, COVID-19 related factors marginally contributed to inequality in mental health among females (0.1%), but remained the second-largest contributor among males (31.1%). In particular, self-reported family member COVID-19 infection accentuated inequality in mental health, explaining 12.9% for females and 4.2% for males, separately. Experiencing food shortage accounted for 11.4% of the inequalities in mental health for females and 20.2% for males. However, engaging in PA reduced inequalities in mental health, accounting for 26.8% of the reduction for females and 7.4% for males.

## Discussion

This study is the first attempt to examine a set of factors affecting SES-related health inequalities and quantify their contributions to health inequalities using data from a nationwide survey in China during the COVID-19 pandemic. We found that the ill SRH in low- and middle-income groups was much higher than those for the high-income group among Chinese adults. The findings were similar to previous studies on income-related inequalities in SRH before the pandemic [12, 14, 19], which showed that high-income groups had better SRH than low-income groups. However, individuals in the high-income group had poorer mental health than those in low- and middle-income groups. In addition, the CCI of ill SRH and mental health were − 0.09 and 0.04, respectively, indicating that ill SRH concentrated among the poor, but ill mental health is more prevalent among the rich. The results on income-related inequalities in SRH are consistent with earlier studies. The pro-poor inequalities in mental health may highlight the magnitude of uncertainties and associated income shocks and lifestyle changes among those better off. One possible explanation is that individuals with high household income are mainly urban residents. The different impacts of COVID-19 on urban and rural areas lead to mental health disparities between the rich and poor. Specifically, during the COVID-19 pandemic, urban residents reported more mental health problems than their rural counterparts in China [30, 31]. There are several possibilities for such discrepancy. First, most confirmed COVID-19 cases in China were reported in urban areas, which resulted in higher sensitivity and vulnerability to psychosocial effects of the pandemic for urban residents. Second, higher population density in urban areas also increased the risk of spread of the virus and gave rise to greater mental health problems, including stress, depression, irritability, insomnia, fear, confusion, anger, frustration, boredom, and stigma associated with quarantine [30, 32]. Finally, social distancing strategies may increase the probabilities of loneliness, isolation, depression, and anxiety [30, 33]. In China, urban residents were generally isolated at home in a relatively confined space, and their daily activities, including working and social activities, were much more affected. In contrast, rural residents were predominantly farmers living in more spacious areas, and their activities and mental health might be less affected by social distancing and quarantine measures [30].

Income is the leading contributor to inequalities in ill SRH and mental health. Previous studies in both developed and developing nations have found similar results [9, 10, 12, 14–16, 18, 19]. One possible explanation is that high-income provides individuals with more opportunities for healthcare services, and it is rather difficult for low-income residents to access essential healthcare services [34, 35]. It is worth highlighting that, although Chinese healthcare system has achieved significant reform, it still presents several major challenges such as financial risk, equalizing benefit across different regions [36–38]. Particularly, the quality of care and efficiency for the individuals with New Rural Cooperative Medical Scheme are very inadequate: The medical assistance programme for poor people simply helps the enrollment in the rural scheme, rather than covering more of their costs. Consequently, access to primary care for poor people has not improved, and financial protection against high healthcare expenses remains quite restricted [36]. Such disparities in healthcare worsen income-related health inequalities. In addition, income disparities may result in differences in other health determinants such as food consumption [19]. Especially during the COVID-19 pandemic, the soaring food price makes it difficult for the poor to obtain adequate food consumption [39].

This study also revealed the important contributions to income-related health inequalities of COVID-19 related variables, particularly PA, self-reported family member COVID-19 infection, experiencing food shortages, and the pandemic severity. One potential explanation is that there are income disparities of these health-related factors. Specifically, compared with the rich, the poor had a disproportionate likelihood of being infected [40, 41]. For example, overcrowding among the disadvantaged would increase the risk of infection [42]. In contrast, people with access to more economic resources have more opportunities to seek medical care and avoid being infected [40]. Although the COVID-19 pandemic is associated with mental health problems, this virus may disproportionately affect populations of different SES [3, 43]. Particular populations might be more vulnerable to the mental health impact of the COVID-19 pandemic, such as individuals residing in areas with high-level severity of the COVID-19 pandemic. Given that the Chinese government implemented fully or partially lockdown policies in regions with high-level pandemic severity (e.g., Wuhan in Hubei province), residents in these regions are more likely to experience food and medication shortage due to insufficient production during COVID-19 lockdown [39, 44]. Moreover, to guarantee social distancing, communities in China strictly restrict individuals’ travel and these residents are more likely to be encouraged not to go out and stay at home. And residents living in regions with high-level pandemic severity are more likely to be worried about themselves and their family members getting COVID-19 infection. As such, these COVID-19 related factors contribute more to income-related health inequalities in regions with high-level pandemic severity. This was also supported by our heterogeneity analysis based on the severity of pandemic, especially for income-related ill-SRH inequalities (group 1: Levels 1–3 pandemic severity residence; group 2: Levels 4 and 5, detailed results are available in Additional files 4 and 5). The vulnerable groups identified by other studies include older adults [3, 45], the homeless [46], migrant workers [47], the mentally ill [48], pregnant women [49], and Chinese students studying abroad [50].

This study has three notable strengths. First, it collected comprehensive information from a large nationwide sample covering 31 province-level administrative units, providing a unique opportunity to assess topics of interest during this critical time. Second, anonymous surveys encourage participation and ensure privacy protection, leading to timely reports and assessment of health conditions during the COVID-19 pandemic. Third, in comparison to gap analysis or Gini coefficients, our use of CI and CCI methodology integrates income into analyzing inequalities in health outcomes.

Nonetheless, this study has limitations. First, because of constraints in resources and the urgency to study COVID-19, we used self-reported health outcomes instead of measured clinical outcomes. Reporting biases thus may exist. Second, as a cross-sectional survey, it cannot track the changes of health inequalities and the contribution of major sources of health inequalities. Third, the data were not nationally representative due to the sampling issue, therefore, the generalization of our key findings should be with much caution. It is worth noting that the average age in our sample was younger (32.0 years old) than that of nationally representative data in China, such as the 2018 China Family Panel Studies (CFPS) (47.9 years old, Additional file 6). In addition, relative to CFPS, the fractions of high-level education and the unmarried were much higher (Additional file 6). And our study was oversampled by urban residents, possibly due to our sampling approach. Especially for pandemic severity in the province of residence, our study sample was relatively comparable to CFPS in Levels 1, 2, and 5. But, for Levels 3 and 4 were much different from those of CFPS (Level 3: 8.06% vs. 17.95% in the CFPS; Level 4: 51.54% vs. 37.95% in the CFPS, Additional file 6). Yet, online surveys provide unique opportunities for research in the COVID-19 era, e.g., many conventional face-to-face surveys are not feasible during the pandemic [51]. Finally, given the relatively small sample sizes of separate analyses, it is difficult for us to perform separate analyses for regions with each different level of severity. Specifically, our full sample is 8448, and the sample size for regions with different levels of pandemic severity is 218 for the Level 1 (only Hubei province), 684 for the Level 2, 681 for the Level 3, 4354 for the Level 4, and 2511 for the Level 5, respectively. Therefore, the explaining power of the regressions may be compromised due to smaller sample sizes for certain regions such as the Level 1. As such, the results from separate analyses may not be reliable and convincing. However, it should be noted that, when estimating the contributions of COVID-19 related variables to the total income-related health inequalities, we have controlled for regions with different levels of pandemic severity.

This study contributes to the understanding of the impacts of modifiable socioeconomic determinants such as income, health insurance, and COVID-19 related factors on SRH and mental health among Chinese adults. These findings have important policy implications. First, the government should continue to strengthen poverty alleviation efforts, thereby mitigating the impact of COVID-19 among the poor. Second, the healthcare system should strive to improve the accessibility of health care services to all populations of different SES, especially in the current pandemics. Public health policies should pay special attention to health inequalities associated with the severity of the pandemic in a region. Finally, we have observed strong effects of food shortage on SRH and mental health. To enhance emergency preparedness, the government should initiate a strategic bottom-up hierarchical food reserve system, starting at the community level and reaching the central government level to provide and coordinate reliable support for residents to reduce food insecurity during pandemics, which would help to reduce income-related health inequalities.

## Conclusions

In conclusion, pro-rich inequalities in SRH and pro-poor inequalities in mental health existed in China during the COVID-19 pandemic. To mitigate health inequality amid the COVID-19 pandemic, policies and interventions need to target the disadvantaged groups, including the poor and those who are severely affected by COVID-19 containment efforts and have experienced hardships in daily life, including food shortage and income loss.

## Supplementary Information

**Additional file 1 **: **Table S1.** The definition of 5 different-level severity areas of COVID-19 pandemic. ^a^ The definition is based on the cumulative number of confirmed cases (*N*) by February 20, 2020.

**Additional file 2 **: **Table S2.** Contribution of each factor to income-related inequalities in ill SRH by gender, the 2020 China COVID-19 Survey. SES = socioeconomic status. CI = Concentration Index of factor k. ^*^ *p* < 0.1, ^**^ *p* < 0.05, ^***^ *p* < 0.01. ^a^ Contribution (%) is defined as the contribution of each factor to the total explained part.

**Additional file 3 **: **Table S3.** Contribution of each factor to income-related inequalities in mental health by gender, the 2020 China COVID-19 survey. CI = Concentration Index of factor k. ^*^ *p* < 0.1, ^**^ *p* < 0.05, ^***^ *p* < 0.01. ^a^ Contribution (%) is defined as the contribution of each factor to the total explained part.

**Additional file 4 **: **Table S4.** Contribution of each factor to income-related inequalities in ill SRH health by different pandemic severity in the province of residence, the 2020 China COVID-19 survey. Notes: CI = Concentration Index of factor k. ^*^ *p* < 0.1, ^**^ *p* < 0.05, ^***^ *p* < 0.01. ^a^ Level I includes provinces of Levels 1–3 and Level II includes Levels 4 and 5. ^b^ Contribution (%) is defined as the contribution of each factor to the total explained part.

**Additional file 5 **: **Table S5**. Contribution of each factor to income-related inequalities in mental health by different pandemic severity in the province of residence, the 2020 China COVID-19 survey. Notes: CI = Concentration Index of factor k. The results are also similar when not controlling pandemic severity in the province of residence in the subsample. ^*^ *p* < 0.1, ^**^ *p* < 0.05, ^***^ *p* < 0.01. ^a^ Level I includes provinces of Levels 1–3 and Level II includes Levels 4 and 5. ^b^ Contribution (%) is defined as the contribution of each factor to the total explained part.

**Additional file 6 **: **Table S6.** Sociodemographic characteristics between the study sample and 2018 China Family Panel Studies (CFPS).

**Additional file 7 **: **Figure S1.** Concentration curve of health. The horizontal line denotes the cumulative share of the population ranked by income, and the vertical line represents the cumulative share of health outcomes. Both dotted lines denote the concentration curves, and the diagonal is defined as the “line of equality.” Area I represents pro-rich health inequality, meaning that better health is concentrated more heavily among the rich. Area II denotes pro-poor health inequality, indicating that better health is concentrated more heavily among the poor.

## Data Availability

The datasets used and/or analysed during the current study are available from the corresponding author on reasonable request.

## Abbreviations

COVID-19: Novel coronavirus disease 2019
CI: Concentration Index
CCI: Corrected Concentration Index
NCDs: Noncommunicable chronic diseases
PA: Physical activity
SES: Socioeconomic status
SRH: Self-reported health
