# Supplementary material for: Income-related health inequality among Chinese adults during the COVID-19 pandemic: evidence based on an online survey
Source: Int J Equity Health. 2021 Apr 26;20:106. doi: 10.1186/s12939-021-01448-9 (PMC8072088; doi:10.1186/s12939-021-01448-9)
Supplement: Supplementary file 1 — Additional file 1 : Table S1. The definition of 5 different-level severity areas of COVID-19 pandemic. a The definition is based on the cumulative number of confirmed cases (N) by February 20, 2020. [file 12939_2021_1448_MOESM1_ESM.docx]

**Table S1.** The definition of 5 different-level severity areas of COVID-19 pandemic

| Level of pandemic severity in the province of residence | Definition ^a^ | Provinces |
| --- | --- | --- |
| Level 1 | *N* ≥ 10,000 | Hubei |
| Level 2 | 1,000 *≤ N* < 10,000 | Guangdong and Zhejiang |
| Level 3 | 500 *≤ N* < 1,000 | Henan, Hunan, Anhui and Jiangxi |
| Level 4 | 100 *≤ N* < 500 | Jiangsu, Chongqing, Shandong, Sichuan, Beijing, Heilongjiang, Shanghai, Fujian, Shaanxi, Hebei, Guangxi, Yunnan, and Hainan |
| Level 5 | *N* < 100 | Liaoning, Shanxi, Tianjin, Jilin, Inner Mongolia, Guizhou, Tibet, Gansu, Ningxia, Xinjiang, and Qinghai |

^a^ The definition is based on the cumulative number of confirmed cases (*N*) by February 20, 2020.
