# Supplementary material for: Income-related health inequality among Chinese adults during the COVID-19 pandemic: evidence based on an online survey
Source: Int J Equity Health. 2021 Apr 26;20:106. doi: 10.1186/s12939-021-01448-9 (PMC8072088; doi:10.1186/s12939-021-01448-9)
Supplement: Supplementary file 2 — Additional file 2 : Table S2. Contribution of each factor to income-related inequalities in ill SRH by gender, the 2020 China COVID-19 Survey. SES = socioeconomic status. CI = Concentration Index of factor k. * p < 0.1, ** p < 0.05, *** p < 0.01. a Contribution (%) is defined as the contribution of each factor to the total explained part. [file 12939_2021_1448_MOESM2_ESM.docx]

**Table S2.** Contribution of each factor to income-related inequalities in ill SRH by gender, the 2020 China COVID-19 Survey

| Variables | Female (n=4,747) | | | Male (n=3,701) | | |
| --- | --- | --- | --- | --- | --- | --- |
|  | Coef. | CI_k_ | Contribution^a^ | Coef. | CI_k_ | Contribution^a^ |
| ***Demographics*** |  |  |  |  |  |  |
| Age (in years) | 0.0052^***^ | 0.0139 | −9.43% | 0.0034^***^ | −0.0036 | 1.71% |
|  |  |  |  |  |  |  |
| ***Socioeconomic status (SES)*** |  |  |  |  |  |  |
| Education |  |  |  |  |  |  |
| Middle | 0.0323 | 0.0506 | −2.76% | 0.1281^***^ | 0.0110 | −2.44% |
| High | 0.0799 | −0.0397 | 6.95% | 0.1668^***^ | −0.0091 | 3.95% |
| Employment status |  |  |  |  |  |  |
| Employed | −0.0268 | 0.0501 | 3.53% | −0.0953^***^ | 0.0254 | 7.61% |
| Student | 0.0275 | −0.2707 | 4.53% | −0.0787^***^ | −0.1063 | −7.37% |
| Retired | −0.0105 | 0.0259 | 0.05% | 0.0352 | 0.1468 | −0.43% |
| Marital status |  |  |  |  |  |  |
| Married/cohabiting | −0.0761^***^ | 0.0647 | 13.97% | −0.0103 | 0.0368 | 1.07% |
| Divorced/separated/widowed | 0.0252 | 0.0408 | −0.09% | 0.0599 | 0.1107 | −0.46% |
| Residence |  |  |  |  |  |  |
| Town | 0.0429^**^ | −0.0282 | 1.30% | −0.0203 | −0.0631 | −1.20% |
| City | 0.0307^*^ | 0.0329 | −2.25% | −0.0364^**^ | 0.0422 | 4.45% |
| Per capita household income last year (continuous) | −0.0003^***^ | 0.7371 | 54.04% | −0.0004^***^ | 0.7478 | 66.94% |
|  |  |  |  |  |  |  |
| ***Chronic diseases (numbers)*** |  |  |  |  |  |  |
| 1 | 0.1126^***^ | −0.0011 | 0.05% | 0.1202^***^ | −0.0219 | 1.24% |
| 2 | 0.1236^***^ | 0.1754 | −4.20% | 0.0488^**^ | 0.1459 | −2.33% |
| ≥3 | 0.0278 | 0.2691 | −1.11% | 0.0611^**^ | 0.1557 | −2.98% |
|  |  |  |  |  |  |  |
| ***Lifestyles*** |  |  |  |  |  |  |
| Alcohol drinking |  |  |  |  |  |  |
| Ex-drinker | 0.0597^**^ | 0.0412 | −0.51% | −0.0186 | −0.0149 | −0.18% |
| Currently drinker | 0.0116 | 0.0686 | −0.38% | 0.0132 | 0.0277 | −0.61% |
| Smoking |  |  |  |  |  |  |
| Ex-smoker | −0.0756^**^ | 0.1814 | 1.82% | 0.0286 | −0.0043 | 0.07% |
| Currently smoker | −0.0909^***^ | 0.2290 | 4.13% | −0.0133 | 0.0802 | 1.46% |
| Knowledge of Dietary Pagoda | −0.0157 | 0.0048 | 0.21% | −0.0594^***^ | 0.0129 | 2.50% |
| Have medical insurance | −0.0142 | −0.0044 | −0.21% | −0.0314^*^ | −0.0016 | −0.20% |
|  |  |  |  |  |  |  |
| ***COVID-19 related variables*** |  |  |  |  |  |  |
| Losing job due to COVID-19 | 0.0184 | 0.0123 | −0.27% | −0.0218^*^ | 0.0418 | 1.64% |
| Self-reported family member COVID-19 infection | −0.1412^***^ | 0.1561 | 5.53% | −0.1172^***^ | 0.1895 | 12.93% |
| Experiencing food shortage during COVID-19 lockdown | 0.0200 | 0.0301 | −0.55% | 0.0120 | 0.0914 | −1.69% |
| Experiencing medication shortage during COVID-19 lockdown | 0.0469^***^ | 0.0174 | −0.84% | 0.0092 | 0.0723 | −1.11% |
| Engaging in any physical activity/exercise during COVID-19 lockdown | −0.1701^***^ | 0.0514 | 20.24% | −0.1334^***^ | 0.0314 | 12.58% |
| Pandemic severity in the province of residence |  |  |  |  |  |  |
| Level 2 pandemic severity | 0.0008 | 0.0047 | 0.00% | −0.0804^**^ | −0.0405 | −1.43% |
| Level 3 pandemic severity | 0.0099 | −0.1268 | 0.38% | −0.0738^**^ | −0.0791 | −2.24% |
| Level 4 pandemic severity | −0.0458 | −0.0125 | −1.18% | −0.0986^***^ | 0.0031 | 0.69% |
| Level 5 pandemic severity | −0.1028^***^ | 0.0561 | 7.05% | −0.1440^***^ | 0.0326 | 5.85% |
| Contribution of COVID-19 related variables |  |  | 30.35% |  |  | 27.22% |
| Total |  |  | 100% |  |  | 100% |

Notes: SRH=self-reported health. CI =Concentration Index of factor k. ^*^ p < 0.1, ^**^ p < 0.05, ^***^ p < 0.01.

^a^ Contribution (%) is defined as the contribution of each factor to the total explained part.
