# Supplementary material for: Income-related health inequality among Chinese adults during the COVID-19 pandemic: evidence based on an online survey
Source: Int J Equity Health. 2021 Apr 26;20:106. doi: 10.1186/s12939-021-01448-9 (PMC8072088; doi:10.1186/s12939-021-01448-9)
Supplement: Supplementary file 3 — Additional file 3 : Table S3. Contribution of each factor to income-related inequalities in mental health by gender, the 2020 China COVID-19 survey. CI = Concentration Index of factor k. * p < 0.1, ** p < 0.05, *** p < 0.01. a Contribution (%) is defined as the contribution of each factor to the total explained part. [file 12939_2021_1448_MOESM3_ESM.docx]

**Table S3.** Contribution of each factor to income-related inequalities in mental health by gender, the 2020 China COVID-19 survey

| Variables | Female (n=4,747) | | | Male (n=3,701) | | |
| --- | --- | --- | --- | --- | --- | --- |
|  | Coef. | CI_k_ | Contribution^a^ | Coef. | CI_k_ | Contribution^a^ |
| ***Demographics*** |  |  |  |  |  |  |
| Age (in years) | −0.0492^***^ | 0.0139 | −22.01% | −0.0758^***^ | −0.0036 | 3.97% |
|  |  |  |  |  |  |  |
| ***Socioeconomic status (SES)*** |  |  |  |  |  |  |
| Education |  |  |  |  |  |  |
| Middle | −0.1570 | 0.0506 | −3.34% | 0.4251 | 0.0110 | 0.85% |
| High | −0.4242 | −0.0397 | 9.19% | 0.2424 | −0.0091 | −0.60% |
| Employment status |  |  |  |  |  |  |
| Employed | 0.0309 | 0.0501 | 1.01% | −0.5757^*^ | 0.0254 | −4.84% |
| Student | −0.7025^**^ | −0.2707 | 28.76% | −0.7519^*^ | −0.1063 | 7.41% |
| Retired | −0.6501^*^ | 0.0259 | −0.70% | −0.4356 | 0.1468 | −0.56% |
| Marital status |  |  |  |  |  |  |
| Married/cohabiting | −0.5562^**^ | 0.0647 | −25.43% | −0.0986 | 0.0368 | −1.08% |
| Divorced/separated/widowed | −1.2617^***^ | 0.0408 | −1.15% | 0.7072 | 0.1107 | 0.57% |
| Residence |  |  |  |  |  |  |
| Town | 0.4012^*^ | −0.0282 | −3.03% | 0.1938 | −0.0631 | −1.21% |
| City | 0.4336^**^ | 0.0329 | 7.91% | 0.1060 | 0.0422 | 1.36% |
| Per capita household income last year (continuous) | 0.0011 | 0.7371 | 46.06% | 0.0020^*^ | 0.7478 | 32.81% |
|  |  |  |  |  |  |  |
| ***Chronic diseases (numbers)*** |  |  |  |  |  |  |
| 1 | 1.3063^***^ | −0.0011 | −0.13% | 1.5228^***^ | −0.0219 | −1.65% |
| 2 | 2.4545^***^ | 0.1754 | 20.75% | 2.4753^***^ | 0.1459 | 12.43% |
| ≥3 | 2.6483^***^ | 0.2691 | 26.39% | 3.0666^***^ | 0.1557 | 15.77% |
|  |  |  |  |  |  |  |
| ***Lifestyles*** |  |  |  |  |  |  |
| Alcohol drinking |  |  |  |  |  |  |
| Ex-drinker | 0.2733 | 0.0412 | 0.58% | 0.3355 | −0.0149 | −0.34% |
| Currently drinker | 0.3777^*^ | 0.0686 | 3.03% | 0.1386 | 0.0277 | 0.67% |
| Smoking |  |  |  |  |  |  |
| Ex-smoker | −0.1450 | 0.1814 | −0.87% | 0.3214 | −0.0043 | −0.08% |
| Currently smoker | 1.0650^***^ | 0.2290 | 12.05% | 0.4488^**^ | 0.0802 | 5.20% |
| Knowledge of Dietary Pagoda | −0.5731^***^ | 0.0048 | −1.94% | −0.4501^**^ | 0.0129 | −1.99% |
| Have medical insurance | −0.7470^***^ | −0.0044 | 2.75% | −0.3126 | −0.0016 | 0.21% |
|  |  |  |  |  |  |  |
| ***COVID-19 related variables*** |  |  |  |  |  |  |
| Losing job due to COVID-19 | 0.7934^***^ | 0.0123 | 2.95% | 1.1801^***^ | 0.0418 | 9.35% |
| Self-reported family member COVID-19 infection | 1.3265^***^ | 0.1561 | 12.93% | 0.3580 | 0.1895 | 4.16% |
| Experiencing food shortage during COVID-19 lockdown | 1.6596^***^ | 0.0301 | 11.42% | 1.3612^***^ | 0.0914 | 20.20% |
| Experiencing medication shortage during COVID-19 lockdown | 0.7080^***^ | 0.0174 | 3.16% | 0.5970^***^ | 0.0723 | 7.59% |
| Engaging in any physical activity/exercise during COVID-19 lockdown | −0.9027^***^ | 0.0514 | −26.75% | −0.7472^***^ | 0.0314 | −7.42% |
| Pandemic severity in the province of residence |  |  |  |  |  |  |
| Level 2 pandemic area | −0.2848 | 0.0047 | −0.09% | −0.4476 | −0.0405 | 0.84% |
| Level 3 pandemic area | −0.8386^*^ | −0.1268 | 7.96% | −0.3727 | −0.0791 | 1.19% |
| Level 4 pandemic area | −0.7013^*^ | −0.0125 | 4.50% | −0.8723^**^ | 0.0031 | −0.64% |
| Level 5 pandemic area | −0.9334^**^ | 0.0561 | −15.95% | −0.9773^**^ | 0.0326 | −4.18% |
| Contribution of Covid-19 related variables |  |  | 0.12% |  |  | 31.08% |
| Total |  |  | 100% |  |  | 100% |

Notes: CI =Concentration Index of factor k. ^*^ p < 0.1, ^**^ p < 0.05, ^***^ p < 0.01.

^a^ Contribution (%) is defined as the contribution of each factor to the total explained part.
