# Supplementary material for: Income-related health inequality among Chinese adults during the COVID-19 pandemic: evidence based on an online survey
Source: Int J Equity Health. 2021 Apr 26;20:106. doi: 10.1186/s12939-021-01448-9 (PMC8072088; doi:10.1186/s12939-021-01448-9)
Supplement: Supplementary file 4 — Additional file 4 : Table S4. Contribution of each factor to income-related inequalities in ill SRH health by different pandemic severity in the province of residence, the 2020 China COVID-19 survey. Notes: CI = Concentration Index of factor k. * p < 0.1, ** p < 0.05, *** p < 0.01. a Level I includes provinces of Levels 1–3 and Level II includes Levels 4 and 5. b Contribution (%) is defined as the contribution of each factor to the total explained part. [file 12939_2021_1448_MOESM4_ESM.docx]

**Table S4.** Contribution of each factor to income-related inequalities in ill SRH health by different pandemic severity in the province of residence, the 2020 China COVID-19 survey

| Variables | Level I pandemic severity residence^a^ | | | Level II pandemic severity residence^a^ | | |
| --- | --- | --- | --- | --- | --- | --- |
|  | Coef. | CI_k_ | Contribution^b^ | Coef. | CI_k_ | Contribution^b^ |
| ***Demographics*** |  |  |  |  |  |  |
| Gender | -0.0449^**^ | 0.0162 | 2.63% | -0.0526^***^ | -0.0015 | -0.14% |
| Age (in years) | 0.0056^***^ | 0.0053 | -6.41% | 0.0043^***^ | 0.0062 | -3.44% |
| ***Socioeconomic status (SES)*** |  |  |  |  |  |  |
| Education |  |  |  |  |  |  |
| Middle | 0.1602 | 0.0331 | -14.31% | 0.0816^**^ | 0.0343 | -4.73% |
| High | 0.2179^**^ | -0.0204 | 19.47% | 0.1268^***^ | -0.0270 | 7.69% |
| Employment status |  |  |  |  |  |  |
| Employed | -0.0936^**^ | 0.0551 | 24.45% | -0.0384^**^ | 0.0350 | 3.73% |
| Student | -0.1096^***^ | -0.1517 | -25.73% | 0.0082 | -0.1984 | 1.07% |
| Retired | -0.0410 | -0.1074 | -0.66% | 0.0149 | 0.0748 | -0.16% |
| Marital status |  |  |  |  |  |  |
| Married/cohabiting | -0.0568^**^ | 0.0814 | 18.59% | -0.0451^***^ | 0.0462 | 5.96% |
| Divorced/separated/widowed | -0.0584 | -0.0969 | -0.64% | 0.0628 | 0.0882 | -0.46% |
| Residence |  |  |  |  |  |  |
| Town | 0.0303 | -0.0378 | 2.04% | 0.0103 | -0.0396 | 0.40% |
| City | 0.0401 | 0.0425 | -7.22% | -0.0141 | 0.0342 | 1.17% |
| Per capita household income last year (continuous) | -0.0003^**^ | 0.7676 | 53.59% | -0.0004^***^ | 0.7356 | 66.82% |
| ***Chronic diseases (numbers)*** |  |  |  |  |  |  |
| 1 | 0.0735^**^ | -0.0731 | 3.33% | 0.1275^***^ | -0.0016 | 0.09% |
| 2 | 0.0675 | 0.1164 | -1.99% | 0.0871^***^ | 0.1562 | -3.58% |
| ≥3 | 0.0699 | 0.2027 | -4.04% | 0.0460^**^ | 0.1933 | -1.96% |
| ***Lifestyles*** |  |  |  |  |  |  |
| Alcohol drinking |  |  |  |  |  |  |
| Ex-drinker | -0.0225 | 0.0812 | 1.24% | 0.0124 | -0.0152 | 0.07% |
| Currently drinker | -0.0083 | 0.0088 | 0.12% | 0.0218^*^ | 0.0456 | -0.92% |
| Smoking |  |  |  |  |  |  |
| Ex-smoker | 0.0262 | 0.1322 | -1.80% | -0.0030 | 0.0272 | 0.02% |
| Currently smoker | -0.0882^***^ | 0.0623 | 5.72% | -0.0231 | 0.1097 | 1.71% |
| Knowledge of Dietary Pagoda | -0.0612^***^ | -0.0060 | -1.85% | -0.0292^***^ | 0.0099 | 0.85% |
| Have medical insurance | -0.0373 | 0.0030 | 0.69% | -0.0180 | -0.0047 | -0.30% |
| ***COVID-19 related variables*** |  |  |  |  |  |  |
| Losing job due to COVID-19 | 0.0130 | 0.0196 | -0.69% | -0.0092 | 0.0292 | 0.37% |
| Self-reported family member COVID-19 infection | -0.1787^***^ | 0.0874 | 5.23% | -0.1400^***^ | 0.1692 | 9.88% |
| Experiencing food shortage during COVID-19 lockdown | -0.0197 | 0.0356 | 1.65% | 0.0195 | 0.0691 | -1.48% |
| Experiencing medication shortage during COVID-19 lockdown | 0.0283 | 0.0016 | -0.11% | 0.0299^***^ | 0.0541 | -2.01% |
| Engaging in any physical activity/exercise during COVID-19 lockdown | -0.1682^***^ | 0.0355 | 25.20% | -0.1548^***^ | 0.0423 | 16.70% |
| Level 2 pandemic severity residence | -0.0521^*^ | 0.0464 | 7.47% |  |  |  |
| Level 3 pandemic severity residence | -0.0356 | -0.0545 | -5.97% |  |  |  |
| Level 5 pandemic severity residence |  |  |  | -0.0534^***^ | 0.0334 | 2.63% |
| Total |  |  | 100% |  |  | 100% |

Notes: CI =Concentration Index of factor k. ^*^ p < 0.1, ^**^ p < 0.05, ^***^ p < 0.01.

^a^ Level I includes provinces of Levels 1-3 and Level II includes Levels 4 and 5.

^b^ Contribution (%) is defined as the contribution of each factor to the total explained part.
