# Supplementary material for: Income-related health inequality among Chinese adults during the COVID-19 pandemic: evidence based on an online survey
Source: Int J Equity Health. 2021 Apr 26;20:106. doi: 10.1186/s12939-021-01448-9 (PMC8072088; doi:10.1186/s12939-021-01448-9)
Supplement: Supplementary file 5 — Additional file 5 : Table S5. Contribution of each factor to income-related inequalities in mental health by different pandemic severity in the province of residence, the 2020 China COVID-19 survey. Notes: CI = Concentration Index of factor k. The results are also similar when not controlling pandemic severity in the province of residence in the subsample. * p < 0.1, ** p < 0.05, *** p < 0.01. a Level I includes provinces of Levels 1–3 and Level II includes Levels 4 and 5. b Contribution (%) is defined as the contribution of each factor to the total explained part. [file 12939_2021_1448_MOESM5_ESM.docx]

**Table S5.** Contribution of each factor to income-related inequalities in mental health by different pandemic severity in the province of residence, the 2020 China COVID-19 survey

| Variables | Level I pandemic severity residence^a^ | | | Level II pandemic severity residence^a^ | | |
| --- | --- | --- | --- | --- | --- | --- |
|  | Coef. | CI_k_ | Contribution^b^ | Coef. | CI_k_ | Contribution^b^ |
| ***Demographics*** |  |  |  |  |  |  |
| Gender | 0.2007 | 0.0162 | 0.94% | -0.1239 | -0.0015 | 0.05% |
| Age (in years) | -0.0815^***^ | 0.0053 | -7.40% | -0.0588^***^ | 0.0062 | -8.16% |
| ***Socioeconomic status (SES)*** |  |  |  |  |  |  |
| Education |  |  |  |  |  |  |
| Middle | -1.7702^*^ | 0.0331 | -12.66% | 0.4306 | 0.0343 | 4.28% |
| High | -1.8373^*^ | -0.0204 | 13.14% | 0.1520 | -0.0270 | -1.58% |
| Employment status |  |  |  |  |  |  |
| Employed | -0.3816 | 0.0551 | -7.98% | -0.0451 | 0.0350 | -0.75% |
| Student | -1.1597^**^ | -0.1517 | 21.79% | -0.4315 | -0.1984 | 9.65% |
| Retired | -1.0799 | -0.1074 | 1.39% | -0.4174 | 0.0748 | -0.75% |
| Marital status |  |  |  |  |  |  |
| Married/cohabiting | -0.3785 | 0.0814 | -9.91% | -0.3223^*^ | 0.0462 | -7.32% |
| Divorced/separated/widowed | 1.2016 | -0.0969 | -1.05% | -0.7410^*^ | 0.0882 | -0.93% |
| Residence |  |  |  |  |  |  |
| Town | 0.3205 | -0.0378 | -1.73% | 0.3057 | -0.0396 | -2.05% |
| City | 0.7105^**^ | 0.0425 | 10.24% | 0.1940 | 0.0342 | 2.77% |
| Per capita household income last year (continuous) | 0.0034^**^ | 0.7676 | 57.76% | 0.0011 | 0.7356 | 29.48% |
| ***Chronic diseases (numbers)*** |  |  |  |  |  |  |
| 1 | 0.4736 | -0.0731 | -1.72% | 1.6216^***^ | -0.0016 | -0.19% |
| 2 | 2.5261^***^ | 0.1164 | 5.96% | 2.4852^***^ | 0.1562 | 17.52% |
| ≥3 | 2.8977^***^ | 0.2027 | 13.40% | 2.8996^***^ | 0.1933 | 21.24% |
| ***Lifestyles*** |  |  |  |  |  |  |
| Alcohol drinking |  |  |  |  |  |  |
| Ex-drinker | 1.2637^***^ | 0.0812 | 5.57% | 0.1274 | -0.0152 | -0.12% |
| Currently drinker | 0.3061 | 0.0088 | 0.36% | 0.2020 | 0.0456 | 1.46% |
| Smoking |  |  |  |  |  |  |
| Ex-smoker | 0.7602 | 0.1322 | 4.18% | 0.0805 | 0.0272 | 0.11% |
| Currently smoker | 0.0090 | 0.0623 | 0.05% | 0.6775^***^ | 0.1097 | 8.59% |
| Knowledge of Dietary Pagoda | -0.6046^**^ | -0.0060 | 1.46% | -0.4728^***^ | 0.0099 | -2.37% |
| Have medical insurance | -0.7795^**^ | 0.0030 | -1.16% | -0.5816^***^ | -0.0047 | 1.66% |
| ***COVID-19 related variables*** |  |  |  |  |  |  |
| Losing job due to COVID-19 | 1.0063^***^ | 0.0196 | 4.25% | 0.9429^***^ | 0.0292 | 6.53% |
| Self-reported family member COVID-19 infection | 0.6914 | 0.0874 | 1.62% | 0.8052^***^ | 0.1692 | 9.75% |
| Experiencing food shortage during COVID-19 lockdown | 1.1048^***^ | 0.0356 | 7.38% | 1.6142 ^***^ | 0.0691 | 20.97% |
| Experiencing medication shortage during COVID-19 lockdown | 0.7898^***^ | 0.0016 | 0.24% | 0.6414^***^ | 0.0541 | 7.40% |
| Engaging in any physical activity/exercise during COVID-19 lockdown | -0.7839^***^ | 0.0355 | -9.40% | -0.8580^***^ | 0.0423 | -15.88% |
| Level 2 pandemic severity residence^c^ | -0.4513 | 0.0464 | -5.18% |  |  |  |
| Level 3 pandemic severity residence | -0.6299^*^ | -0.0545 | 8.46% |  |  |  |
| Level 5 pandemic severity residence |  |  |  | -0.1627 | 0.0334 | -1.37% |
| Total |  |  | 100% |  |  | 100% |

Notes: CI =Concentration Index of factor k. The results are also similar when not controlling pandemic severity in the province of residence in the subsample. ^*^ p < 0.1, ^**^ p < 0.05, ^***^ p < 0.01.

^a^ Level I includes provinces of Levels 1-3 and Level II includes Levels 4 and 5.

^b^ Contribution (%) is defined as the contribution of each factor to the total explained part.
