# Supplementary material for: Income-related health inequality among Chinese adults during the COVID-19 pandemic: evidence based on an online survey
Source: Int J Equity Health. 2021 Apr 26;20:106. doi: 10.1186/s12939-021-01448-9 (PMC8072088; doi:10.1186/s12939-021-01448-9)
Supplement: Supplementary file 6 — Additional file 6 : Table S6. Sociodemographic characteristics between the study sample and 2018 China Family Panel Studies (CFPS). [file 12939_2021_1448_MOESM6_ESM.docx]

**Table S6.** Sociodemographic characteristics between the study sample and 2018 China Family Panel Studies (CFPS)

| Variables | Study sample  Mean (SD) /*N* (%) | CFPS 2018  Mean (SD) /*N* (%) |
| --- | --- | --- |
| Gender |  |  |
| Male | 3,701 (43.81%) | 14,135 (49.54%) |
| Female | 4,747 (56.19%) | 14,400 (50.46%) |
| Age (in years) | 32.04 (9.97) | 47.92 (17.42) |
| Education |  |  |
| Low | 163 (1.93%) | 12,708 (44.53%) |
| Middle | 3,479 (41.18%) | 14,271 (50.01%) |
| High | 4806 (56.89%) | 1,556 (5.45%) |
| Employment status |  |  |
| Unemployed | 1,207 (14.29%) | 7,075 (24.79%) |
| Employed | 7,241 (85.71%) | 21,460 (75.21%) |
| Marital status |  |  |
| Unmarried | 2,508 (29.69%) | 2,327 (8.15%) |
| Married/cohabiting | 5,774 (68.35%) | 23,854 (83.60%) |
| Divorced/separated/widowed | 166 (1.96%) | 2,354 (8.25%) |
| Residence |  |  |
| Rural | 1,282 (15.18%) | 14,082 (49.35%) |
| Urban | 7,166 (84.82%) | 14,453 (50.65) |
| Pandemic severity in the province of residence |  |  |
| Level 1 pandemic severity | 218 (2.58%) | 416 (1.46%) |
| Level 2 pandemic severity | 684 (8.10%) | 3,336 (11.69%) |
| Level 3 pandemic severity | 681 (8.06%) | 5,122 (17.95%) |
| Level 4 pandemic severity | 4,354 (51.54%) | 10,828 (37.95%) |
| Level 5 pandemic severity | 2,511 (29.72%) | 8,833 (30.95%) |
| Obs. | 8,448 | 28,535 |
