# Supplementary material for: Income-related health inequality among Chinese adults during the COVID-19 pandemic: evidence based on an online survey
Source: Int J Equity Health. 2021 Apr 26;20:106. doi: 10.1186/s12939-021-01448-9 (PMC8072088; doi:10.1186/s12939-021-01448-9)
Supplement: Supplementary file 7 — Additional file 7 : Figure S1. Concentration curve of health. The horizontal line denotes the cumulative share of the population ranked by income, and the vertical line represents the cumulative share of health outcomes. Both dotted lines denote the concentration curves, and the diagonal is defined as the “line of equality.” Area I represents pro-rich health inequality, meaning that better health is concentrated more heavily among the rich. Area II denotes pro-poor health inequality, indicating that better health is concentrated more heavily among the poor. [file 12939_2021_1448_MOESM7_ESM.docx]

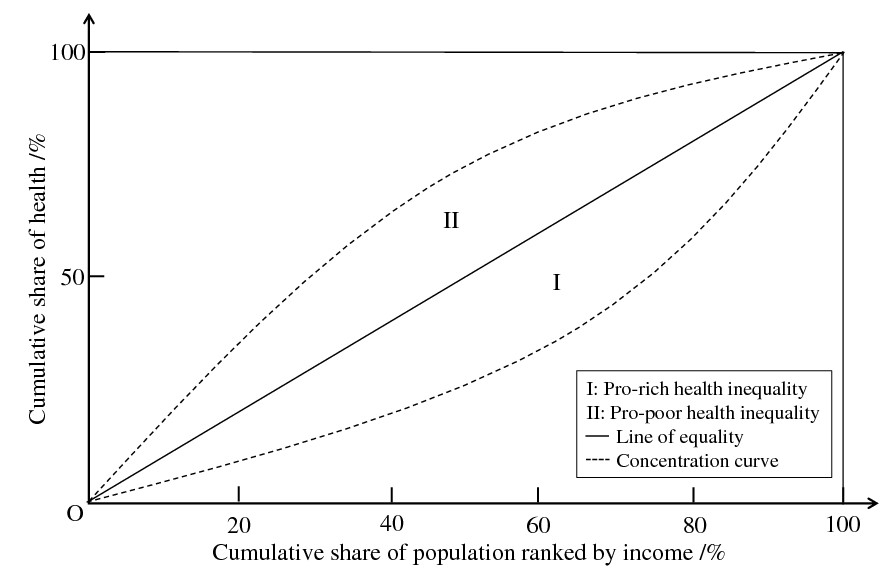


**Figure S1.** Concentration curve of health

Notes: The horizontal line denotes the cumulative share of the population ranked by income, and the vertical line represents the cumulative share of health outcomes. Both dotted lines denote the concentration curves, and the diagonal is defined as the “line of equality.” Area I represents pro-rich health inequality, meaning that better health is concentrated more heavily among the rich. Area II denotes pro-poor health inequality, indicating that better health is concentrated more heavily among the poor.
